# Zinc asparaginate supplementation induces redistribution of toxic trace elements in rat tissues and organs

**DOI:** 10.1515/intox-2015-0020

**Published:** 2015-09

**Authors:** Andrey A. Skalny, Alexey A. Tinkov, Yulia S. Medvedeva, Irina B. Alchinova, Mikhail Yu. Karganov, Olga P. Ajsuvakova, Anatoly V. Skalny, Alexandr A. Nikonorov

**Affiliations:** 1 Federal State Scientific Institution “Institute of Toxicology”, Federal Medico-Biological Agency, Bekhtereva str. 1, St. Petersburg 192019, Russia; 2 Russian Society of Trace Elements in Medicine, ANO “Centre for Biotic Medicine”, Zemlyanoy Val St. 46, Moscow 105064, Russia; 3 Laboratory of Biotechnology and Applied Bioelementology, Yaroslavl State University, Sovetskaya st., 14, Yaroslavl, 150000, Russia; 4 Department of Biochemistry, Orenburg State Medical University, Sovetskaya St., 6, Orenburg, 460000, Russia; 5 Laboratory of Physicochemical and Ecological Pathophysiology, Institute of General Pathology and Pathophysiology, Baltiyskaya St., 8, Moscow, 125315, Russia; 6 Department of Chemistry and Methods of Chemistry Teaching, Orenburg State Pedagogical University, Sovetskaya st., 19, Orenburg, 460014, Russia; 7 Institute of Bioelementology (Russian Satellite Centre of Trace Element – Institute for UNESCO), Orenburg State University, Pobedy Ave. 13, Orenburg 460352, Russia

**Keywords:** zinc, metal distribution, toxic trace elements, antagonism, lithium

## Abstract

The primary objective of the current study was the investigation of the influence of zinc asparaginate supplementation for 7 and 14 days on toxic metal and metalloid content in rat organs and tissues. Rats obtained zinc asparaginate in doses of 5 and 15 mg/kg/day for 7 and 14 days. At the end of the experiment rat tissues and organs (liver, kidney, heart, m. gastrocnemius, serum, and hair) were collected for subsequent analysis. Estimation of Zn, Al, As, Li, Ni, Sn, Sr content in the harvested organs was performed using inductively coupled plasma mass spectrometry at NexION 300D. The obtained data showed that intragastric administration of zinc significantly increased liver, kidney and serum zinc concentrations. Seven-day zinc treatment significantly affected the toxic trace element content in the animals’ organs. Zinc supplementation significantly decreased particularly liver aluminium, nickel, and tin content, whereas lead tended to increase. Zinc-induced changes in kidney metal content were characterized by elevated lithium and decreased nickel concentration. Zinc-induced alteration of myocardical toxic element content was multidirectional. Muscle aluminium and lead concentration were reduced in response to zinc supplementation. At the same time, serum and hair toxic element concentrations remained relatively stable after 7-day zinc treatment. Zinc asparaginate treatment of 14 days significantly depressed liver and elevated kidney lithium content, whereas a significant zinc-associated decrease was detected in kidney strontium content. Zinc supplementation for 14 days resulted also in multidirectional changes in the content of heart toxic elements. At the same time, significant zinc-associated decrease in muscle lithium and nickel levels was observed. Fourteen-day zinc treatment resulted in significantly increased serum arsenic and tin concentrations, whereas hair trace element content remained relatively stable. Generally, the obtained data indicate a significant redistribution of toxic metals in the animal organism under zinc supplementation.

## Introduction

The intensive development of heavy industry resulted in increased emission of heavy metals into the environment (Nriagu, 1996). Heavy metal exposure is associated with a number of diseases, like cardiovascular pathology (Alissa & Ferns, 2011), obesity and diabetes mellitus (Hyman, 2010), and neurodegeneration (Jomova *et al*., 2010). The main mechanisms of metal toxicity are activation of freeradical oxidation and inflammation (Valko *et al*., 2005).

Zinc is an essential metal involved in a number of cellular processes. The efficiency of various zinc compounds against certain diseases is currently investigated (Matsukura & Tanaka, 2000; Sakurai & Adachi, 2005; Oliveira *et al*., 2006). Zinc possesses antioxidant activity due to its structural role in antioxidant systems and the ability to protect protein sulfhydryl groups from oxidation (Powell, 2000). An anti-inflammatory potential of zinc-containing compounds has also been demonstrated (Prasad, 2008). Consequently, zinc may be considered a functional antagonist of toxic metals. However, data on the influence of zinc on toxic trace element distribution in the organism are contradictory (Goyer, 1997).

Consequently, the primary objective of the current study was the investigation of the influence of zinc asparaginate supplementation for 7 and 14 days on toxic metal and metalloid content in organs and tissues of the rat.

## Materials and methods

### Study design

The experiment was performed in accordance with animal ethics regulations. The protocol has been approved by the Local Ethics Committee. Male Wistar rats (No.36) with equal body weight were used in the current investigation. The animals were acclimatized to laboratory conditions for 14 days. They were maintained under cyclic lighting (12h light/dark cycle). The rats were fed a standard laboratory chow PK-120 for laboratory animals (Laboratorkorm Ltd., Moscow, Russia) with total caloric content of 307 kcal/100 g. Data on diet trace element content obtained in our laboratory are presented in Table 1.

**Table 1:** Trace element content in the laboratory chow.

Element	Content (μg/g)
Zn	78.59±5.09
Al	68.11±3.52
As	0.35±0.01
Li	0.13±0.01
Ni	2.66±0.03
Sn	0.01±0.00
Sr	72.13±0.96

Data expressed as Mean ± SD

The first group of animals was used as the control one. Zinc asparaginate (Zn(C_4_NO_4_H_6_)_2_∙Zn(OH)_2_) was administered intragastrically at doses of 5 and 15 mg/kg/day for animals in the respective 2^nd^ (ZnA_5_) and 3^rd^ (ZnA_15_) group. Intragastric administration was performed using a silicone catheter after mixing the agent with starch.

At the end of the experiment, tissues and organs (liver, kidney, heart, m. gastrocnemius, serum, and hair) were collected for subsequent analysis. Parenchimatous organs were separated from connective tissue and rinsed with ice-cold physiological saline.

### Sample preparation and chemical analysis

Hair samples were washed with acetone and rinsed twice with deionized water for removal of exogenous contamination (Zhao *et al*., 2012) with subsequent drying on air at 60 °C. Serum was diluted with an acidified solution (pH = 2.0; 1:15, v/v) containing 1% 1-butanol (Merck KGaA, Darmstadt, Germany), 0.1% Triton X-100 (Sigma-Aldrich, Co., St. Louis, USA) and 0.07% HNO_3_ (Sigma-Aldrich, Co., St. Louis, USA) in distilled deionized water.

All samples were degraded in a Berghof speedwave four system (Berghof, Products Instruments GmbH, Eningen, Germany). The amount of 50 mg of each tissue was placed into Teflon tubes containing concentrated nitric acid. The digestion was performed for 20 minutes at 180 °C.

Chemical analysis of the obtained solutions was performed by inductively-coupled plasma mass-spectrometry at NexION 300D (PerkinElmer Inc., Shelton, CT 06484, USA) equipped with an automated sampler ESI SC-2 DX4 (Elemental Scientific Inc., Omaha, NE 68122, USA) and using Dynamic Reaction Cell technology for removal of the majority of interferences without loss of sensitivity.

The preparation of the system was performed according to the manufacturer's recommendations. The system was calibrated using standard trace element solutions with final concentrations of 0.5, 5, and 50 μg/l, prepared from the commercially available Universal Data Acquisition Standards Kit (PerkinElmer Inc., Shelton, CT 06484, USA) by dilution with acidified distilled deionized water. Internal standardization of the analysis was performed using 10 μg/l yttrium isotope (^89^Y) solution prepared from Pure Single-Element Standard (PerkinElmer Inc., Shelton, CT 06484, USA). The matrix containing 8% 1-butanol (Merck KGaA, Darmstadt, Germany), 0.8% Triton X-100 (Sigma-Aldrich, Co., St. Louis, USA), 0.02% tetramethylammonium hydroxide (Alfa-Aesar, Ward Hill, USA), and 0.02% ethylenediaminetetraacetic acid (Sigma-Aldrich, Co., St. Louis, USA) was used for preparation of the yttrium solution. Laboratory quality control was performed using reference materials. Commercially available hair GBW09101 (Shanghai Institute of Nuclear Research, Shanghai, China) and ClinCheck Plasma Control lot 129, 1 and 2 levels (RECIPE Chemicals + Instruments GmbH, Germany) were used as reference materials for hair and serum analysis, respectively. Recovery rates for all metals studied exceeded 80% for both hair and serum.

### Statistical analysis

The obtained data were treated with Statistica 10 (StatSoft Inc., Tulsa, USA). Statistical analysis indicated that data distribution was not Gaussian. The obtained group values were therefore expressed as median and the respective 25 and 75 percentiles. The significance of the overall tendency was assessed using Kruskal-Wallis test. Comparison of group values was performed by the means of Mann-Whitney U-test. All differences were considered to be significant at *p*<0.05.

## Results

### The influence of zinc supplementation on tissue zinc distribution

Intragastric zinc administration altered metal distribution in organs and tissues of the rats (Table 2). In particular, treatment with 15 mg/kg/day zinc asparaginate for 7 days resulted in a significant 15% elevation of liver zinc content. At the same time, the overall tendency was not significant. Serum zinc concentration in groups 2 and 3 was 25% and 33% higher in comparison to the control values. The overall trend was also significant in accordance with Kruskal-Wallis analysis.

**Table 2:** Zinc content in rats organs and tissues.

Tissue	Control	ZnA_5_	ZnA_15_	*p*-value
**7 days**				
Liver	30.3 (24.4–31.5)	30.9 (30.4–34.0)	34.8 (31.4–40.1) ^1^	0.104
Muscle	9.7 (6.7–10.2)	8.7 (7.8–10.0)	10.1 (8.1–12.9)	0.593
Kidney	18.7 (18.2–19.9)	18.1 (17.5–18.6)	20.1 (19.4–21.4) ^2^	0.453
Heart	17.0 (16.4–17.9)	16.5 (15.8–18.4)	16.4 (16.0–16.5) ^1^	0.291
Serum	1.2 (0.9–1.4)	1.5 (1.4–1.8) ^1^	1.6 (1.6–1.8) ^1^	0.048*
Hair	149.0 (143.0–159.0)	152.0 (146.0–154.0)	147.5 (136.0–151.0)	0.413
**14 days**				
Liver	28.8 (26.4–29.8)	29.8 (28.7–31.0)	34.4 (31.4–36.5) ^1,2^	0.012*
Muscle	10.0 (9.8–12.3)	12.3 (10.3–13.5)	10.4 (8.1–18.6)	0.785
Kidney	17.1 (16.7–18.9)	18.7 (18.4–18.9)	20.1 (19.0–21.7) ^1^	0.214
Heart	15.9 (15.4–16.7)	16.3 (16.1–16.8)	16.5 (15.6–17.2)	0.476
Serum	0.9 (0.8–1.0)	1.5 (1.1–1.7) ^1^	1.9 (1.6–2.1) ^1^,2	0.002*
Hair	123.2 (116.8–130.5)	137.5 (131.2–138.5)	138.1 (127.8–150.2)	0.139

Data expressed as Median (25–75)

1 – Significant difference in comparison to Control (I) animals (*p*<0.05)

2 – Significant difference in comparison to ZnA5 (II) animals (*p*<0.05)

* – *p*-trend is significant at *p*-values <0.05.

Treatment of 14-days was more effective in modification of the zinc status. Particularly, liver zinc levels in rats obtaining 15 mg/kg/day zinc asparaginate significantly exceeded the respective values obtained for groups 1 and 2 by respective 19% and 15%. Serum zinc concentration in rats treated with 5 and 15 mg/kg/day zinc asparaginate was by respective 66% and 111% higher than the control values. The overall tendency was significant in both cases. Zinc treatment significantly increased kidney zinc content in animals from the 3^rd^ group by 18% as compared to control values. However, the overall tendency was not significant.

### Effect of 7-day zinc asparaginate treatment on distribution of toxic trace elements

Seven-day zinc treatment significantly affected toxic metal and metalloid content of the animals’ organs (Table 3). In particular, intragastric administration of zinc asparaginate in the dose of 15 mg/kg/day decreased liver concentration of aluminium, nickel, and tin more than 3-, 2.5-, and 5-fold, respectively. Moreover, the overall tendency to zinc-associated alteration of toxic element content was significant in accordance with Kruskal-Wallis analysis. At the same time, zinc supplementation resulted in 66% increase in liver lead content in rats from the 3^rd^ group. However, the general trend was not significant. On the contrary, 7-day zinc treatment resulted in more than 2 and 3-fold increase in kidney lithium content in rats from the 3^rd^ group in comparison to the respective group 1 and 2 values. The overall tendency to increased kidney lithium level was also significant. At the same time, kidney nickel content in animals obtaining 5 and 15 mg/kg/day was significantly decreased by respective 32 and 27% in comparison to the control values. Zinc-induced alteration of myocardical toxic element content was multidirectional. In particular, intragastric administration of 15 mg/kg/day zinc asparaginate resulted in a significant 2-fold increase in heart lithium levels as compared to the 1^st^ and 2^nd^ group values. Heart muscle lead content in the 3^rd^ group was 75% higher than that in the 1^st^ and 2^nd^ group. The overall tendency was significant in both cases. At the same time, zinc treatment significantly depressed heart nickel levels more than twofold. Animals obtaining 15 mg/kg/day zinc asparaginate were also characterized by 25% lower values of myocardial strontium content as compared to control values.

**Table 3:** Influence of 7-day intragastric administration of zinc asparaginate on toxic element content in rat organs (μg/g).

Element	Control	ZnA_5_	ZnA_15_	*p*-value
Liver Al	0.967 (0.922–1.917)	0.800 (0.555–1.384)	0.320 (0.289–0.411) ^1^	0.018*
Liver As	0.127 (0.108–0.150)	0.147 (0.136–0.159)	0.143 (0.136–0.163)	0.368
Liver Li	0.005 (0.004–0.005)	0.004 (0.004–0.004)	0.005 (0.004–0.005)	0.281
Liver Ni	0.019 (0.017–0.021)	0.014 (0.013–0.020)	0.007 (0.006–0.009) ^1^	0.049*
Liver Pb	0.003 (0.002–0.003)	0.004 (0.003–0.007)	0.005 (0.004–0.006) ^1^	0.055
Liver Sn	0.011 (0.004–0.025)	0.005 (0.004–0.022)	0.002 (0.001–0.003) ^1,2^	0.003*
Liver Sr	0.042 (0.039–0.045)	0.063 (0.040–0.069)	0.039 (0.038–0.043)	0.140
Kidney Al	0.423 (0.399–0.505)	0.309 (0.246–.375)	0.478 (0.351–0.968)	0.117
Kidney As	0.061 (0.061–0.065)	0.073 (0.061–0.081)	0.070 (0.064–0.078)	0.098
Kidney Li	0.005 (0.004–0.005)	0.003 (0.003–0.004) ^1^	0.011 (0.009–0.011) ^1,2^	0.001*
Kidney Ni	0.037 (0.032–0.041)	0.025 (0.021–0.026) ^1^	0.027 (0.022–0.034) ^1^	0.013*
Kidney Pb	0.004 (0.002–0.008)	0.004 (0.003–0.004)	0.006 (0.005–0.009)	0.172
Kidney Sn	0.002 (0.002–0.003)	0.002 (0.001–0.008)	0.003 (0.002–0.003)	0.907
Kidney Sr	0.094 (0.087–0.104)	0.087 (0.084–0.094)	0.089 (0.086–0.100)	0.796
Heart Al	0.410 (0.395–0.571)	0.586 (0.491–0.721)	0.392 (0.341–0.674)	0.700
Heart As	0.065 (0.061–0.078)	0.056 (0.044–0.067)	0.061 (0.056–0.065)	0.281
Heart Li	0.003 (0.003–0.004)	0.003 (0.003–0.004)	0.007 (0.007–0.007) ^1,2^	0.003*
Heart Ni	0.025 (0.020–0.026)	0.012 (0.010–0.014) ^1^	0.012 (0.011–0.013) ^1^	0.025*
Heart Pb	0.004 (0.003–0.005)	0.004 (0.003–0.004)	0.007 (0.005–0.008) ^2^	0.031*
Heart Sn	0.004 (0.002–0.011)	0.003 (0.003–0.004)	0.002 (0.002–0.002)	0.078
Heart Sr	0.060 (0.058–0.069)	0.051 (0.045–0.055)	0.045 (0.043–0.051) ^1^	0.011*

Data expressed as Median(25–75)

1 – Significant difference in comparison to Control (I) animals (*p*<0.05)

2 – Significant difference in comparison to ZnA5 (II) animals (*p*<0.05)

* - *p*-trend is significant at *p*-values <0.05.

Intragastric administration of 15 mg/kg/day zinc asparaginate significantly decreased skeletal muscle aluminium content by 49 and 65% in comparison to the respective 1^st^ and 2^nd^ group values (Table 4). A significant tendency to zinc-associated lead depression was also observed. The concentration of serum toxic elements remained relatively stable after zinc treatment. Significant changes were observed only in the case of serum tin levels. Seven-day zinc asparaginate treatment significantly decreased the hair arsenic content. In particular, the hair arsenic content in rats obtaining 15 mg/kg/day zinc asparaginate was 16% lower than that in the control group. Hair nickel levels in groups 2 and 3 were characterized by more than 4- and 6-fold decrease in comparison to the control values. The overall tendency to zinc-associated decrease in hair arsenic and nickel was significant in accordance with Kruskal-Wallis analysis.

**Table 4:** Influence of 7-day intragastric administration of zinc asparaginate on tissue (μg/g) and serum (μg/l) toxic element levels in rats.

Element	Control	ZnA_5_	ZnA1_5_	*p*-value
Muscle Al	0.770 (0.660–1.937)	1.121 (0.822–1.586)	0.395 (0.338–0.429) ^1,2^	0.004*
Muscle As	0.011 (0.009–0.014)	0.010 (0.009–0.010)	0.010 (0.009–0.012)	0.546
Muscle Li	0.006 (0.005–0.007)	0.005 (0.004–0.005)	0.005 (0.005–0.005)	0.331
Muscle Ni	0.030 (0.029–0.035)	0.027 (0.019–0.032)	0.031 (0.017–0.038)	0.796
Muscle Pb	0.008 (0.007–0.011)	0.004 (0.003–0.005) ^1^	0.006 (0.005–0.007) ^2^	0.016*
Muscle Sn	0.003 (0.002–0.004)	0.004 (0.003–0.013)	0.002 (0.001–0.002) ^2^	0.087
Muscle Sr	0.070 (0.058–0.088)	0.061 (0.053–0.063)	0.068 (0.066–0.073) ^2^	0.143
Serum Al	0.030 (0.010–0.120)	0.026 (0.013–0.104)	0.014 (0.011–0.019)	0.480
Serum As	0.013 (0.010–0.031)	0.012 (0.011–0.019)	0.011 (0.010–0.012)	0.489
Serum Li	0.002 (0.001–0.002)	0.002 (0.001–0.002)	0.002 (0.002–0.002)	0.479
Serum Ni	0.004 (0.002–0.007)	0.003 (0.002–0.003)	0.002 (0.002–0.002)	0.432
Serum Pb	0.001 (0.000–0.001)	0.001 (0.000–0.002)	0.000 (0.000–0.001)	0.237
Serum Sn	0.000 (0.000–0.000)	0.000 (0.000–0.001)	0.000 (0.000–0.000) ^2^	0.035*
Serum Sr	0.141 (0.116–0.168)	0.113 (0.102–0.128)	0.119 (0.114–0.139)	0.2662
Hair Al	1.550 (1.080–3.490)	1.360 (0.842–4.290)	0.946 (0.755–1.400)	0.372
Hair As	0.026 (0.024–0.028)	0.025 (0.022–0.026)	0.021 (0.020–0.023) ^1,2^	0.044*
Hair Li	0.024 (0.022–0.025)	0.023 (0.022–0.024)	0.016 (0.014- 0.022)	0.117
Hair Ni	0.460 (0.211–1.160)	0.116 (0.099–0.131) ^1^	0.074 (0.058–0.101) ^1^	0.042*
Hair Pb	0.037 (0.022–0.064)	0.059 (0.028–0.089)	0.024 (0.015–0.044)	0.372
Hair Sn	0.015 (0.012–0.026)	0.051 (0.005–0.216)	0.010 (0.007–0.016)	0.598
Hair Sr	0.647 (0.557–0.713)	0.759 (0.610–1.060)	0.564 (0.511–0.730)	0.444

Data expressed as Median (25–75)

1 – Significant difference in comparison to Control (I) animals (*p*<0.05)

2 – Significant difference in comparison to ZnA5 (II) animals (*p*<0.05)

* - *p*-trend is significant at *p*-values <0.05.

### Effect of 14-day zinc asparaginate treatment on toxic trace element distribution

Zinc asparaginate treatment significantly depressed liver lithium content (Table 5). In particular, liver Li levels in groups 2 and 3 were significantly lower than the control values by 29% and 57%, respectively, The overall trend to decreased lithium content in the liver of rats was also significant in accordance with Kruskal-Wallis test. On the contrary, a significant increase in kidney lithium content was detected in response to zinc treatment. Particularly intragastric administration of 5 and 15 mg/kg/day zinc asparaginate enhanced kidney lithium content by 25% and 60%, respectively. Kidney strontium content in rats obtaining 15 mg/kg/day zinc was 10% lower than that of control animals. The overall trend to zinc-induced decrease in kidney strontium content was also significant. As observed after 7-day treatment, zinc supplementation for 14 days resulted in multidirectional changes in heart toxic element content. Intragastric gavage of 15 mg/kg/day zinc asparaginate significantly increased myocardial arsenic content by 25% and 49% as compared to the respective values of groups 1 and 2. Rats from group 3 were characterized by 40% and 25% lower values of myocardial lithium content in comparison to the values of group 1 and 2. The overall tendency to zinc-associated decrease in heart nickel level was also significant.

**Table 5:** Influence of 14-day intragastric administration of zinc asparaginate on toxic element content in rat organs (μg/g).

Element	Control	ZnA_5_	ZnA_15_	*p*-value
Liver Al	0.660 (0.360–0.818)	0.406 (0.366–0.507)	0.296 (0.244–0.400)	0.148
Liver As	0.099 (0.093–0.115)	0.076 (0.067–0.090)	0.090 (0.075–0.100)	0.104
Liver Li	0.007 (0.006–0.009)	0.005 (0.005–0.005) ^1^	0.003 (0.002–0.003) ^1,2^	<0.001*
Liver Ni	0.015 (0.009–0.023)	0.009 (0.008–0.011)	0.009 (0.009–0.011)	0.386
Liver Pb	0.004 (0.003–0.008)	0.004 (0.003–0.006)	0.003 (0.003–0.004)	0.423
Liver Sn	0.004 (0.003–0.005)	0.003 (0.002–0.005)	0.003 (0.002–0.005)	0.425
Liver Sr	0.040 (0.038–0.048)	0.042 (0.039–0.042)	0.043 (0.037–0.048)	0.977
Kidney Al	0.271 (0.231–0.333)	0.284 (0.273–0.302)	0.293 (0.229–0.328)	0.960
Kidney As	0.048 (0.045–0.055)	0.043 (0.039–0.045)	0.047 (0.038–0.055)	0.679
Kidney Li	0.004 (0.004–0.005)	0.005 (0.005–0.006) ^1^	0.006 (0.006–0.007) ^1,2^	0.007*
Kidney Ni	0.030 (0.023–0.034)	0.027 (0.025–0.032)	0.030 (0.022–0.035)	0.986
Kidney Pb	0.004 (0.003–0.005)	0.004 (0.003–0.004)	0.003 (0.003–0.005)	0.960
Kidney Sn	0.002 (0.002–0.003)	0.002 (0.001–0.002)	0.002 (0.001–0.002)	0.291
Kidney Sr	0.082 (0.075–0.089)	0.082 (0.080–0.085)	0.074 (0.068–0.077) ^2^	0.040*
Heart Al	0.333 (0.245–0.360)	0.295 (0.287–0.357)	0.287 (0.243–0.338)	0.520
Heart As	0.051 (0.050–0.057)	0.043 (0.039–0.046) ^1^	0.064 (0.061–0.071) ^1,2^	<0.001*
Heart Li	0.005 (0.004–0.006)	0.004 (0.004–0.005)	0.003 (0.002–0.004) ^1,2^	0.012*
Heart Ni	0.013 (0.011–0.018)	0.006 (0.005–0.007) ^1^	0.007 (0.006–0.010)	0.021*
Heart Pb	0.005 (0.004–0.005)	0.004 (0.003–0.004)	0.003 (0.002–0.004)	0.291
Heart Sn	0.001 (0.001–0.001)	0.002 (0.001–0.002)	0.003 (0.001–0.003)	0.402
Heart Sr	0.047 (0.037–0.049)	0.046 (0.041–0.050)	0.045 (0.038–0.045)	0.581

Data expressed as Median (25–75)

1 – Significant difference in comparison to Control (I) animals (*p*<0.05)

2 – Significant difference in comparison to ZnA5 (II) animals (*p*<0.05)

* - *p*-trend is significant at *p*-values <0.05.

Despite the absence of significant differences in group values, the overall tendency to zinc-associated decrease in skeletal muscle lithium content was significant (Table 6). Intragastric administration of 5 and 15 mg/kg/day zinc asparaginate significantly decreased muscle nickel content by 52% and 59% in comparison to the respective group values. A significant 4-fold increase in serum arsenic content was detected in animals treated with 15 mg/kg/day zinc asparaginate in comparison with group 1 and 2 values. Moreover, a significant tendency to zinc-associated increase in serum tin levels was observed. Hair trace element content remained relatively stable in response to 14-day zinc treatment. Significant changes were observed only in the case of strontium. In particular, hair strontium content in groups 2 and 3 was by respective 34% and 55% lower than that in the control group.

**Table 6:** Influence of 14-day intragastric administration of zinc asparaginate on tissue (μg/g) and serum (μg/l) toxic element levels in rats.

Parameter	Control	ZnA_5_	ZnA_15_	*p*-value
Muscle Al	0.443 (0.388–0.574)	0.346 (0.311–0.605)	0.335 (0.291–0.565)	0.399
Muscle As	0.008 (0.006–0.008)	0.008 (0.007–0.009)	0.005 (0.005–0.005) ^2^	0.109
Muscle Li	0.005 (0.004–0.005)	0.006 (0.006–0.007) ^1^	0.005 (0.004–0.005) ^2^	0.008*
Muscle Ni	0.027 (0.018–0.031)	0.013 (0.011–0.014) ^1^	0.011 (0.009–0.015) ^1^	0.021*
Muscle Pb	0.008 (0.006–0.012)	0.004 (0.003–0.007)	0.004 (0.003–0.008)	0.164
Muscle Sn	0.009 (0.004–0.018)	0.004 (0.002–0.007)	0.003 (0.002–0.005) ^1^	0.164
Muscle Sr	0.069 (0.061–0.079)	0.061 (0.048–0.064)	0.058 (0.051–0.064)	0.250
Serum Al	0.114 (0.058–0.152)	0.098 (0.087–0.118)	0.111 (0.046–0.153)	0.927
Serum As	0.007 (0.006–0.009)	0.007 (0.005–0.013)	0.028 (0.021–0.037) ^1,2^	0.008*
Serum Li	0.002 (0.001–0.002)	0.001 (0.001–0.002)	0.002 (0.002–0.002)	0.264
Serum Ni	0.004 (0.003–0.005)	0.003 (0.003–0.004)	0.004 (0.004–0.006)	0.263
Serum Pb	0.001 (0.000–0.001)	0.000 (0.000–0.001)	0.001 (0.000–0.001)	0.347
Serum Sn	0.000 (0.000–0.000)	0.000 (0.000–0.000)	0.000 (0.000–0.001) ^1^	0.036*
Serum Sr	0.161 (0.140–0.179)	0.160 (0.152–0.178)	0.161 (0.146–0.170)	0.994
Hair Al	1.196 (0.882–1.456)	0.769 (0.544–0.957)	1.258 (0.816–2.055)	0.476
Hair As	0.017 (0.016–0.017)	0.015 (0.014–0.017)	0.016 (0.012–0.018)	0.431
Hair Li	0.017 (0.015–0.017)	0.017 (0.016–0.019)	0.012 (0.010–0.014)	0.148
Hair Ni	0.053 (0.046–0.059)	0.053 (0.033–0.058)	0.042 (0.022–0.114)	0.927
Hair Pb	0.028 (0.025–0.173)	0.033 (0.027–0.043)	0.055 (0.040–0.433)	0.324
Hair Sn	0.038 (0.018–0.684)	0.039 (0.017–0.049)	0.069 (0.046–0.168)	0.191
Hair Sr	0.827 (0.727–1.234)	0.545 (0.396–0.578)	0.372 (0.351–0.400)	0.003*

Data expressed as Median (25–75)

1 – Significant difference in comparison to Control (I) animals (*p*<0.05)

2 – Significant difference in comparison to ZnA5 (II) animals (*p*<0.05)

* - *p-*trend is significant at *p-*values <0.05.

## Discussion

The obtained data demonstrate a redistribution of toxic trace elements in the rat organism in response to zinc asparaginate treatment (Table 7). In particular, decreased liver metal and metalloid content may be indicative of their decreased content in the organism as the liver is a central organ in metal homeostasis. The results of the present study are partially in agreement with previous data indicating a decrease in liver nickel content in animals consuming zinc-containing drinking water (Sidhu *et al*., 2004). It has been also shown that tin and zinc are antagonists in respect to their liver concentration (Johnson & Greger, 1984). A previous study demonstrating lithium-induced decrease in liver zinc content (Tandon *et al*., 1999) conforms to the results of the present study. Moreover, the fact of modulation of lithium-induced hepatotoxicity using zinc (Chadha *et al*., 2008) also supports the idea of antagonism between zinc and lithium in liver. Similar results were obtained for aluminium (Bhasin *et al*., 2014).

**Table 7:** Summary of the effects of zinc on toxic element content in rat tissues and organs (based on the obtained Kruskal-Wallis *p*-values).

Tissue	Liver		Kidney		Heart		Muscle		Serum		Hair	
Days	7	14	7	14	7	14	7	14	7	14	7	14
Al	↓	↔	↔	↔	↔	↔	↓	↔	↔	↔	↓	↔
As	↔	↔	↔	↔	↔	↑	↔	↔	↔	↑	↔	↔
Li	↔	↓	↑	↑	↑	↓	↔	↓	↔	↔	↔	↔
Ni	↓	↔	↓	↔	↓	↓	↔	↓	↔	↔	↓	↔
Pb	↔	↔	↔	↔	↑	↔	↓	↔	↔	↔	↔	↔
Sn	↓	↔	↔	↔	↔	↔	↔	↔	↓	↑	↔	↔
Sr	↔	↔	↔	↓	↓	↔	↔	↔	↔	↔	↔	↓

↓ - decreased level; ↑ - ↓ncreased level; ↔ - no significant changes in metal concentration

Increased kidney lithium content along with its decreased level in other studied tissues may be indicative of its increased excretion through urine, taking into account especially the primary role of kidneys in its excretion (Thomsen & Schou, 1968).

It is notable that serum concentration of certain metals increased in response to zinc treatment. We hypothesize that this phenomenon may occur due to zinc-medicated mobilization of heavy metals from their complexes in tissues of the body.

At the same time, some toxic trace elements were characterized by interesting patterns of distribution. In particular, 14-day zinc treatment resulted in increased serum and heart arsenic content. We hypothesize that such elevation may be caused by elimination of As from other depots of the organism that are different from the organs studied, as liver, kidney, muscle content of arsenic is not affected by the treatment. Nearly the same situation was observed in the case of tin. A significant increase in serum Sn concentration without alteration of the content in other tissues also indicates that tin mobilizes from other organs where labile pool of the element is present. An interesting redistribution in two types of muscles (skeletal and heart) was observed in the case of lead. Hypothetically, zinc is able to displace lead ions from its complexes with biological ligands in muscles but not in the myocardium. The intimate mechanisms mediating such redistribution are however unknown.

Relative stability of hair trace element content may be a consequence of short duration of the experiment. In particular, we suppose that the duration was not sufficient to provide metal incorporation into the hair matrix.

The exact mechanisms involved in zinc-induced metal redistribution are unknown. We suppose that the main mechanism of the observed effects is metallonthioneine-dependent. Metallothioneine is a cysteine-rich protein (Vasak & Meloni, 2011) involved in toxic metal detoxication (Rojesijadi, 2000). Particularly zinc has been shown to play a significant role in metallothioneine production (Maret, 2000). The dependence of metallothioneine content in tissue on the duration of metal exposure (Bernotiene *et al*., 2013) may at least partially explain the difference in toxic element status observed between 7 and 14 days. At the same time, the different rate of metallothioneine synthesis in tissues may also affect distribution of toxic elements (Onosaka & Cherian, 1981).

Finally, interaction between zinc and toxic elements at the stage of intestinal absorption may also take place (Peraza *et al*., 1998) and result in decreased heavy metal entrance into the organism.

At the same time, a number of chemical properties of the metals studied may explain the observed interplay between zinc and toxic metals.

Zinc and nickel are elements of the same period and electronic family (3d-metals). The hydrolysis and complexation reactions are rather characteristic for nickel and for zinc. A large number of different geometry complexes are known for Ni (II) (Ribas *et al*., 1999; Maldanis *et al*., 2002; Bagihalli *et al*., 2008; Angelusiu *et al*., 2010) due to the combination of steric and electronic effects. Ni^2+^ ion acts as a substituting agent for Zn^2+^ in its bio-complexes (Rezlescu *et al*., 2000) due to the similarity of ionic radius (0.089 nm for Zn^2+^; 0.083 nm for Ni^2+^) and most frequent coordination numbers (4, 5, 6). Thus the capability to substitute each other in coordination compounds is the pesumable basis of Zn (II) and Ni (II) antagonism.

Aluminum (III) ion is a strong Lewis acid and electron acceptor because of free electron p-orbital (Krahl *et al*., 2006). Al(III) is considered to be a typical complexing agent. Consequently, a very slow exchange rate of water molecules of the first coordination sphere is characteristic for aqua cation [Al(H_2_O)6]^3+^ (Kowall *et al*., 1998). Consequently, aluminum ions are prone to substitution of double-charged cations *(e.g.* calcium and magnesium) due to the similarity of coordination numbers, ionic radiuses and ionization potentials. Complex stability for one and the same ligand depends on central ion electronegativity (Beck & Nagypal, 1989). Thus, it is expected that in the case of double-charged zinc cation this process is reversible and Zn^2+^ (electronegativity by Pauling scale is 1.65) replaces Al^3+^ (electronegativity by Pauling scale is 1.61) because zinc forms more stable bio-complexes (Emsley, 1989).

Both arsenic ([Ar]3d^10^4s^2^4p^3^) and zinc ([Ar]3d^10^4s^2^) are the elements of the end of the 4 period. The primary oxidation degrees for As are +3 or +5 in its compounds with other elements (Cotton *et al*., 1999). At the same time, complex formation with sulfur-containing ligands (e.g. sulfide ion) being soft bases is characteristic for arsenic. Consequently, arsenic toxicity is usually explained by its ability to form strong covalent bonds with sulfur atoms of protein sulfhydryl groups (Albert, 1985). Zinc in its turn is also prone to form thiolate cluster complex with cysteine (Vallee & Auld, 1990; Maret & Vallee, 1998; Zou *et al*., 2002). Thus the antagonism of zinc and arsenic may be probably explained by the competition for ligands like cysteine.

It is most likely that _38_Sr [Ar]5S^2^ exists in the form of insoluble salts (hydroxophosphates) in bones (Oliveira *et al*., 2012) or in the form of the complex with bioligands (Wiesbrock *et al*., 2002; Solov'ev *et al*, 2006; Agostinho *et al*., 2008) in the organism. The mechanism of strontium ion substitution by zinc ion can be explained from the view of conception of strong and soft acids and bases. Strontium cation, like its group analogues (calcium and magnesium), is referred to as strong acid, whereas zinc is a more soft acid. Consequently, it may be proposed that zinc decreases strontium levels due to its displacement from Sr complexes with nitrogen and sulfur donor atoms (soft bases).

Pb(II) and Sn(II) ions also form stable complexes with amino acids and many other bioligands containing RSand HS-functional groups (Shindo & Brown, 1965; Xin & Pope, 1996). The relatively high ionic radius of Pb(II) and Sn(II) allows to attribute these ions to the group of soft acids. Therefore, antagonism between Zn^2+^, Sn^2+^ and Pb^2+^ ions can be explained as in the case of strontium. Moreover, higher second ionization potential for Zn^2+^ (1733 kJ/mol) compared with Sn^2+^ and Pb^2+^ (1411 and 1450 kJ/mol respectively (Emsley, 1989)) indicates higher stability of zinc complexes than in the case of tin and lead. Therefore, zinc may displace Sn^2+^ and Pb^2+^ ions from their complexes with bioligands due to the formation of more stable coordination compounds.

From all of the metals studied, the system “lithiumzinc” is the most problematic for supposition of the chemical mechanisms of the observed antagonism. At the same time, due to weak complexing properties of lithium, one can exclude the possibility of substitution of lithium ions from complexes with bioligands by zinc.

Generally, the obtained data indicate a significant redistribution of toxic metals in the animal organism under zinc supplementation. At the same time, further studies are required to elucidate the intimate mechanisms of such interactions.
